# Reversal of Diabesity Through Modulating Sympathetic Inputs to Adipose Tissue Following Carotid Body Resection

**DOI:** 10.1111/apha.70074

**Published:** 2025-06-24

**Authors:** Bernardete F. Melo, Joana F. Sacramento, Julien Lavergne, Fátima O. Martins, Daniela Rosendo‐Silva, Clara Panzolini, Cláudia S. Prego, Aidan Falvey, Elena Olea, Paulo Matafome, Asuncion Rocher, Jesus Prieto‐Lloret, Miguel C. Correia, Phillipe Blancou, Silvia V. Conde

**Affiliations:** ^1^ iNOVA4Health, NOVA Medical School, Faculdade de Ciências Médicas Universidade Nova de Lisboa Lisboa Portugal; ^2^ CNRS, Institut de Pharmacologie Moléculaire et Cellulaire Université Côte D'azur Valbonne France; ^3^ Institute of Clinical and Biomedical Research (iCBR), Faculty of Medicine and CIBB University of Coimbra Coimbra Portugal; ^4^ Clinical‐Academic Center of Coimbra Coimbra Portugal; ^5^ Departamento de Enfermeria Universidad de Valladolid Valladolid Spain; ^6^ Unidad de Excelencia Instituto de Biomedicina y Genética Molecular (IBGM), Consejo Superior de Investigaciones Científicas Universidad de Valladolid Valladolid Spain; ^7^ Polytechnic University of Coimbra, Coimbra Health School Coimbra Portugal; ^8^ Departamento de Bioquímica, Biologia Molecular y Fisiologia Universidad de Valladolid Valladolid Spain

**Keywords:** adipose tissue, carotid body, catecholaminergic signaling, energy expenditure, metabolic diseases, sympathetic nervous system

## Abstract

**Background and Aims:**

The development of innovative strategies to treat diabesity and its comorbidities is of major societal importance. The carotid bodies (CB), classically defined as O_2_ sensors, are also metabolic sensors whose dysfunction contributes to the genesis and progression of metabolic disturbances. Here, we tested the hypothesis that the CBs are key players in the neural hypothalamic‐sympathetic circuit controlling glucose and energy homeostasis. Moreover, we investigated if abolishment of CB activity has an anti‐diabesity effect in Wistar rats and C75BL/6J mice, associated with increased visceral white and brown adipose tissue (AT) metabolism and the restoration of sympathetic activity within these tissues.

**Results:**

We demonstrate that resection of the carotid sinus nerve, the CB‐sensitive nerve, promotes weight loss and restores metabolic function in obese rats and mice by enhancing tyrosine hydroxylase expression at the paraventricular nucleus of the hypothalamus and its efferent sympathetic neurons to the AT. Moreover, we found that CSN resection increases sympathetic integration and catecholaminergic action in the AT in a manner that restores or even increases AT metabolism.

**Conclusion:**

We provide groundbreaking and innovative data showing a new circuit involving the CB‐hypothalamus‐sympathetic efferents and the AT in controlling glucose and energy homeostasis and so a novel pathway for managing diabesity.

## 1 Introduction

Diabesity, a global epidemic characterized by the co‐occurrence of obesity and type 2 diabetes, represents a significant public health concern [1]. This condition, along with its associated comorbidities such as metabolic‐associated fatty liver disease, obstructive sleep apnea, and cardiovascular diseases, contributes substantially to global mortality [1]. Given its status as a major health emergency and the substantial challenges it poses in disease management, coupled with the fact that current therapies are still predominantly based on lifestyle modifications or non‐personalized pharmacological treatments that lack long‐term effectiveness, there is an urgent need for new pathophysiological mechanisms and therapeutic innovations in this area.

The carotid bodies (CB) are peripheral chemoreceptors that respond to hypoxia by increasing chemosensory activity in the carotid sinus nerve (CSN), causing hyperventilation and activation of the sympathoadrenal system [2]. Furthermore, CBs are also metabolic sensors implicated in the regulation of peripheral insulin sensitivity, glucose homeostasis, and lipid metabolism. Notably, CB chemosensory activity is increased in prediabetes and type 2 diabetes animal models and overweight prediabetic patients [2, 3]. CB ablation through resection or electrical modulation of the CSN has been shown to prevent and/or reverse the metabolic disturbances caused by hypercaloric diets in rats [2, 4, 5, 6]. This improvement is associated with enhanced insulin signaling and glucose uptake in the liver and visceral white adipose tissue (WAT), along with reduced weight gain [4]. These findings suggest a role for the CB in regulating adipose tissue metabolism.

The evidence also suggests that the CB facilitates whole‐body sympathetic activity [7, 8, 9] contributing to metabolic dysregulation [2, 4, 10, 11]. Another piece of evidence indicating CB‐regional/tissue‐specific activation of the sympathetic nervous system (SNS) is that activation of CB by hypoxia inhibits sympathetic outflow to brown adipose tissue (BAT) [12]. Moreover, sympathetic innervation of the adipose tissue modulates lipolysis and thermogenesis via the release of norepinephrine (NE) [13] and dopamine release [14]. Therefore, in the present manuscript, we hypothesize that the CB may also regulate WAT and BAT metabolism through the modulation of the sympathetic nervous system. The CB signals reach the brain via integration in the nucleus tractus solitarius [15], and it was recently described that CB inputs are also integrated in the paraventricular nucleus (PVN) of the hypothalamus [16], one of the most important autonomic control nuclei in the brain regulating metabolism and energy balance [17]. Furthermore, inhibition of tyrosine hydroxylase (TH)‐positive neurons in the PVN resulted in increased blood glucose, and their activation improved glucose clearance [18]. Herein, we hypothesized that the CBs are key players in the neural circuit governing glucose and energy homeostasis and that the CB afferent inputs play a critical role in coordinating sympathetic neural circuits controlling adipose tissue metabolism.

In brief, our findings demonstrate that CB afferent inputs modulate sympathetic drive to both brown and white adipose tissue with the consequent modulation of their metabolism. Moreover, we show that the beneficial effects of CSN resection on weight gain and dysmetabolism are associated with heightened TH levels at the PVN of the hypothalamus and the reconfiguration and enhancement of CB‐mediated sympathetic inputs to adipose tissue. This discovery identifies a novel therapeutic pathway for the management of diabesity.

## 2 Material and Methods

### 2.1 Diets and Animal Care

Experiments were performed in 8–9 weeks‐old male Wistar rats (200–300 g) obtained from the animal facility of the NOVA Medical School, Faculty of Medical Sciences, Universidade NOVA de Lisboa, Portugal, and 4‐week‐old male C75BL/6J mice purchased from Charles River (Lyon, France) and accommodated at the animal facility of the Institut de Pharmacologie Moléculaire et Cellulaire, Université Cotê de Azur, Valbonne, France. After randomization, the 8–10 animals (rats and mice, respectively) were assigned to a HF group fed a 60% fat diet (61.6% fat from which 36% was saturated, 41% monounsaturated, and 23% polyunsaturated, 20.3% carbohydrate, and 19.1% protein; Test Diets, Missouri, USA) for 10 weeks (Wistar rats) or 12 weeks (C75BL/6J mice) or to an aged‐matched control group fed with a standard diet (7.4% fat from which 0.6% was saturated, 0.7% monounsaturated and 2.1% polyunsaturated, 75% carbohydrates, of which 4% was sugar and 17% protein). After 10 and 12 weeks of diet for rats and mice, respectively, both HF and control groups were randomly divided, and half of the group was submitted to CSN resection and the other half submitted to sham procedure, in which the CSN was left intact. These procedures were performed as previously described by Sacramento et al. [4].

After CSN resection, the groups were maintained under the respective diets for 9 weeks and 3 weeks for rats and mice, respectively, as the CSN regrowth period differs between the two species. During all the experimental period animals were caged in pairs and kept under temperature and humidity control (21°C ± 1°C; 55% ± 10% humidity) with a 12 h light/12 h dark cycle and were given ad libitum access to food and water, except during the night prior to insulin sensitivity and glucose tolerance evaluation. Body weight was monitored weekly. Energy and liquid intake were monitored daily, and the caloric intake was calculated before and after the CSN resection. Insulin sensitivity and glucose tolerance were also evaluated throughout the experimental period. CSN resection was confirmed by the abolishment of responses to hypoxia in conscious, freely moving animals by plethysmography.

In a terminal experiment, the animals were anesthetized with sodium pentobarbitone (60 mg/kg, i.p), and catheters were placed in the femoral artery for arterial blood pressure measurement. Blood was collected by cardiac puncture for quantification of serum and plasma of mediators (such as insulin and C‐peptide levels) and the adipose tissue pads (visceral, perinephric, epididymal and subcutaneous depots) were then rapidly collected, weighed, and stored at either −80°C or transferred to 4% PFA or collected and maintained in Tyrode medium (140 mM NaCl, 5 mM KCl, 2 mM CaCl_2_, 1.1 mM MgCl_2_, 10 mM Hepes and 5.5 mM glucose; pH 7.4) on ice for O_2_ consumption rate (OCR) analysis.

Laboratory care was in accordance with the European Union Directive for Protection of Vertebrates Used for Experimental and Other Scientific Ends (2010/63/EU). The experimental protocols were approved by the NOVA Medical School/Faculdade de Ciências Médicas Ethics Committee (n°08/2015/CEFCM and 41/2021/CEFCM), by the Portuguese Direção‐Geral de Alimentação e Veterinária (DGAV 0421/000/000/2016 and 0421/000/000/2021) and by the National Ethical Committee of France (CIEPAL #2018082016528754). Sample size calculation was performed at https://www.calculator.net/sample‐size‐calculator.html and based on previous experiments using hypercaloric diets and CSN resection [2, 3, 4, 5, 6]. For the calculations, we assumed a moderate effect size (Cohen's *f* ≈ 0.25), a significance level (*α*) of 0.05, and a statistical power (1–*β*) of 0.80 (80%). Note also that not all experimental procedures and analyses, such as western blot analysis of protein levels, were performed on mice. This decision was made to avoid a significant increase in the number of animals required, given the small amount of adipose tissue available from each mouse.

### 2.2 Insulin Sensitivity and Glucose Tolerance Evaluation

Insulin sensitivity was evaluated using an insulin tolerance test (ITT) after overnight fasting (Wistar rats) or 5 h fasting (C75BL/6J mice) as previously described [6]. In brief, fasting blood glucose was measured and immediately followed by an insulin bolus (100 mU/kg for Wistar rats or 0.5 U/Kg for C75BL/6J mice), administered via the tail vein, for Wistar rats or intraperitoneally, for C75BL/6J mice. Subsequently, the decline in plasma glucose concentration was measured over a 15 min period with 1 min interval in the rats or at minutes 5, 10, 20, 30, 45, 60, 90, and 120, after insulin administration, in the mice, after insulin administration. Glucose tolerance was evaluated by oral glucose tolerance test (OGTT) after overnight fasting followed by administration of a saline solution containing glucose (2 g/kg or 1.5 g/kg, for rats or mice, respectively; VWR Chemicals, Leuven, Belgium) by gavage, as described [6]. Blood was collected via tail tipping, and glucose levels were measured with a glucometer (Precision Xceed Meter, Abbott Diabetes Care, Portugal) and test strips (Abbott Diabetes Care, Portugal).

### 2.3 Pletismography

Ventilation was measured in conscious freely moving rats as previously described by Melo et al. [6]. The system (emka Technologies, Paris, France) consisted of 5‐L methacrylate chambers continuously perfused with gases (2 L/min). Tidal volume (VT; mL), respiratory frequency (f; breaths/min; bpm), the product of these two variables, and minute ventilation (VE; mL/min/Kg) were monitored. Each rat was placed in the plethysmography chamber and allowed to breathe room air for 30 min until it had adapted to the chamber environment and acquired a standard resting behavior. The protocol consisted in animal acclimatization during 30 min followed by 10 min of normoxia (20% O_2_ balanced N_2_), 10 min of hypoxia (10% O_2_ balanced N_2_), 10 min of normoxia, 10 min of hypercapnia (20% O_2_ + 5% CO_2_ balanced N_2_), and finally to 10 min of normoxia. A high‐gain differential pressure transducer allows measurements of the pressure changes within the chamber reflecting VT. False pressure changes due to rat movement were rejected. Pressure signals were transmitted to a computer for visualization and storage for later analysis using emka software (emka Technologies, Paris, France).

### 2.4 Evaluation of Autonomic Nervous System

The balance between the sympathetic and parasympathetic components of the autonomic nervous system was made by calculating the sympathetic nervous system (SNS) and parasympathetic nervous system (PNS) indexes, calculated in the Kubios HRV software (www.kubios.com). The SNS index in Kubios is based on mean heart rate, Baevsky's stress index, and low frequency power expressed in normalized units, and the PNS index, which is based on the mean intervals between successive heartbeats (RR intervals), the root mean square of successive RR interval differences (RMSSD) and high frequency power expressed in normalized units. Heart rate and RR intervals were obtained using Iox 2.9.5.73 software (Emka Technologies, Paris, France), with an acquisition frequency of 500 Hz.

### 2.5 Quantification of Biomarkers: Plasma Insulin, C‐Peptide, Lipid Profile, Catecholamines, and Glycerol

Insulin and C‐peptide concentrations were determined using an enzyme‐linked immunosorbent assay kit (Mercodia Ultrasensitive Rat Insulin ELISA Kit and Mercodia Rat C‐peptide ELISA Kit, respectively, Mercodia AB, Uppsala, Sweden). Catecholamines were measured in plasma and in homogenized perienteric and BAT samples by HPLC with electrochemical detection as previously described [19]. The lipid profile was assessed using a RANDOX kit (RANDOX, Irlandox, Porto, Portugal). For glycerol quantification, as an index of lipolysis, rat visceral adipose tissue samples were collected from the animals and incubated for 120 min in the presence of isoproterenol (10 μM) (Sigma‐Aldrich, Madrid, Spain). The glycerol released into the media was quantified using a glycerol colorimetric assay (Glycerol assay kit, Sigma‐Aldrich, Madrid, Spain).

### 2.6 Western Blot Analysis

Visceral WAT (100 mg) and BAT (75 mg) depots were homogenized in Zurich medium (10 mM Tris–HCl, 1 mM EDTA, 150 mM NaCl, 1% Triton X‐100, 1% sodium cholate, 1% SDS) with a protease inhibitor cocktail. The samples were centrifuged (Eppendorf, Madrid, Spain), and the supernatant was collected and frozen at −80°C until further use. Samples of the homogenates and the pre‐stained molecular weight markers (Precision, BioRad, Madrid, Spain) were separated by SDS electrophoresis and electro‐transferred to polyvinylidene difluoride membranes (0.45 μM, Millipore, Spain). After 1 h of blocking in milk, the membranes were incubated overnight at 4°C with the primary antibodies against β2 receptors (1:200; 47 kDa; Alomone, Jerusalem, Israel), β3 receptors (1:200; 45KDa; Alomone, Jerusalem, Israel), D1R (1:200; 48 kDa; Abcam, Cambridge, UK), D2R (1:200; 49 kDa, Sigma‐Aldrich, Madrid, Spain), Dopamine β hydroxylase (DβH) (1:1000; Merck, Darmstadt, Germany), HSL (1:1000; 83 KDa; Cell Signaling Technology, Massachusetts, EUA), pAMPK (phospho Thr172) (1:1000; 60 kDa; Cell Signaling Technology, Massachusetts, EUA), pATGL (phospho S406) (1:1000; 55 kDa; Abcam, Cambridge, UK), PGC‐1α (1:1000; 92 kDa; Santa Cruz Biotechnology INC, Texas, EUA), PPARγ (1:1000; 53–57 kDa, Cell Signaling Technology, Massachusetts, EUA), TH (1:1000; 60 kDa; Abcam, Cambridge, UK). The membranes were washed with Tris‐buffered saline with Tween (TBST) (0.1%) and incubated with rabbit anti‐goat (1:5000; Thermofisher Scientific, Massachusetts, EUA), goat anti‐mouse (1:5000; Bio‐Rad Laboratories, California, EUA) or goat anti‐rabbit (1:5000; Bio‐Rad Laboratories, California, EUA) in TBS and developed with enhanced chemiluminescence reagents (ClarityTM Western ECL substrate, Hercules, CA, USA). The intensity of the signals was detected in a Chemidoc Molecular Imager (Chemidoc; BioRad, Madrid, Spain) and quantified using Image Lab software (BioRad). The membranes were re‐probed and tested for Calnexin (1:1000; 90 kDa; SICGEN, Cantanhede, Portugal) and/or β‐actin (1:1000; 42 kDa; SICGEN, Cantanhede, Portugal) immunoreactivity to compare and normalize the expression of proteins with the amount of protein loaded.

### 2.7 Histological and Immunohistochemical Evaluation of WAT

Visceral WAT and BAT depots were collected, dissected, and immersion fixed in 4% PFA. The samples were then embedded into paraffin (Sakura Finetek Europe B.V., Zoeterwoude, The Netherlands) and serial longitudinal sections of 8 or 10 μm thickness were obtained using a Microtome Microm HM200 (MICROM Laborgeräte GmbH, ZEISS Group, Walldorf, Germany).

For evaluation of adipocyte perimeter, after sectioning, the samples were transferred to slides after sectioning and stained with hematoxylin and eosin to stain the nuclei, extracellular matrix, and cytoplasm. Representative photographs were acquired using NDP.view2 software (Hamamatsu, Japan) in slides digitally scanned in the Hamamatsu NanoZoomerSQ (Hamamatsu, Japan). Adipocytes perimeter was visualized using software Fiji app for Image J (https://imagej.nih.gov/ij/).

UCP1 protein and mitochondrial density were evaluated by immunohistochemistry. Paraffin sections were deparaffinized and rehydrated, followed by antigen retrieval and blocking, performed with a 5% bovine serum albumin solution for 60 min. Sections were then incubated with the primary antibody rabbit anti‐UCP1 (1:1000 for WAT and BAT; Santa Cruz Biotechnology INC, Texas, EUA), overnight at 4°C. The sections were then incubated with anti‐rabbit secondary antibody Alexa 488 (1:4000 for WAT and 1:6000 for BAT; Termofisher Scientific, Massachusetts, EUA), Mitotracker Red CMXRos [15 nM] for WAT and [1 nM] for BAT (Termofisher Scientific, Massachusetts, EUA), and DAPI (1 μg/mL; Santa Cruz Biotechnology INC, Texas, EUA) for 90 min. Finally, the section slides were mounted with Dako mounting medium (Agilent, California, EUA), visualized using a Z2 Zeiss widefield Microscope (ZEISS Group, Walldorf, Germany) and analyzed using the Fiji app for Image J software (https://imagej.nih.gov/ij/).

### 2.8 Evaluation of the Oxygen Consumption Rate

Three weeks after CSN resection, mice were sacrificed by cervical dislocation, and visceral WAT and BAT were collected for oxygen consumption rate (OCR) assessment using the Seahorse XF24 (Seahorse Bioscience, North Billerica, MA). Tissue samples were placed in an XF24 Islet Capture Microplate (Seahorse Bioscience, North Billerica, MA) and once in position, were rinsed twice with Seahorse XF DMEM assay medium (Seahorse Bioscience, North Billerica, MA) supplemented with 10 mM glucose, 1 mM pyruvate, and 2 mM L‐glutamine. Finally, 575 μL of assay medium was added to all sample and control wells. Before the measurement of the OCR, the microplate was incubated at 37°C without CO_2_ for 45 min. To evaluate adrenergic stimulation, OCR was measured after the application of norepinephrine [15 μM] (Sigma‐Aldrich, Lisboa, Portugal) or dopamine [100 nM] (Sigma‐Aldrich, Lisboa, Portugal). OCR was calculated by plotting the O_2_ tension of the media as a function of time (pmol/min).

### 2.9 In Vivo Tissue‐Specific Glucose Uptake Evaluation

An intravenous glucose tolerance test (IVGTT) was performed in sham versus CSN–transected normal chow and HF animals. For that, the animals were fasted overnight and a bolus of 2‐deoxy‐D‐[1,2‐3H]‐glucose (1 mC/mL; specific activity: 20 Ci/mmol; PerkinElmer, Madrid, Spain) mixed with glucose (100 μCi/kg body weight; 0.5 g/kg body weight) was administered in the tail vein. Blood samples were taken from the tail vein at regular intervals (0, 2, 5, 10, 15, 30, and 60 min).

To determine glucose‐specific activity, 20 μL plasma was deproteinized with 200 μL of ice‐cold perchloric acid (0.4 N) and centrifuged, and radioactivity was measured in a scintillation counter (Tri‐Carb 2800TR, Perkin‐Elmer, Madrid, Spain). After 60 min, the animals were euthanized, and the tissues (white and brown adipose tissue depots) were rapidly excised. 2‐deoxy‐D‐[^3^H]glucose incorporation was measured in 50–200 mg tissue homogenized in 1 mL of ice cold perchloric acid (0.4 N). The samples were centrifuged, and the radioactivity in the supernatant was measured in a scintillation counter and analysed as previously described [4].

### 2.10 Light‐Sheet Based Fluorescent Microscopy

After perfusion, the excised WAT was washed with PBS and clarified using the iDisco+ method (https://idisco.info/). Briefly, samples were dehydrated at room temperature in successive washes of 20% metanol (MetOH) for 1 h, 40% MetOH for 1 h, 60% MetOH for 1 h, 80% MetOH for 1 h, 100% MetOH for 1 h, and 100% MetOH overnight. Samples were then incubated in a solution of 33% MetOH and 66% Di‐ChloroMethan (DCM, Sigma) overnight and washed twice with 100% methanol for 1 h. Samples were then bleached in chilled fresh 5% H_2_O_2_ in methanol overnight, at 4°C, before rehydration with methanol/H_2_O series (80%, 60%, 40%, 20%, and PBS, 1 h each at room temperature). The samples were then immunolabeled with anti‐TH (AB152, Merck, France) for 24 h after overnight permeabilization at 37°C and overnight blocking with 1.7% TritonX‐ 100, 6% donkey serum, and 10% DMSO in PBS. After three washes in PBS/0.2% Tween‐20, samples were incubated with donkey anti‐goat secondary antibody (Jackson Immunoresearch, Cambridgeshire, UK) for 24 h. Samples were then dehydrated at room temperature in successive baths as described above and then incubated in a solution of 33% MetOH and 66% DCM for 3 h at RT, then in 100% DCM twice for 15 min each, and transferred overnight into the clearing medium 100% DiBenzylEther 98% (Sigma).

Imaging was performed using a homemade light‐sheet ultramicroscope. The samples were placed in a cubic cuvette filled with DBE and placed on the Z‐stage of the bench. It was illuminated with planar sheets of light formed by cylindrical lenses. Light from a multi‐wavelength (561 nm) laser bench (LBX‐4C, Oxxius) was coupled into the setup via two single‐mode optical fibers, allowing illumination from one or two sides. Two‐sided illumination was used. The specimen was imaged from above with a MVX10 macroscope, through a PlanApo 2X/0.5 NA objective (Olympus) with an additional zoom of the macroscope of 1.6, which was oriented perpendicular to the 561 nm light sheet. Images were captured using a sCMOS camera (Orca‐Flash4.0) synchronized with the z‐stage moving the sample through the light sheet. The ultramicroscope is controlled using Micro‐manager software and z‐stacks of images were taken every 2 μm. The image stacks were merged using the alpha‐blending method with a homemade ImageJ macro (Rasband, W.S., ImageJ, U.S. National Institutes of Health, Bethesda, Maryland, USA, http://imagej.nih.gov/ij/, 1997e2012).

### 2.11 Free‐Floating Tyrosine Hydroxylase Immunohistochemistry in the Paraventricular Nucleus of the Hypothalamus

Cryopreserved brains of sham vs. CSN‐transected NC and HF animals were cut in coronal sections (40 μm) using a cryostat and collected for a 6‐well plate with 4 sections/well in PBS. Coordinates used for sectioning were −2.16 mm and −1.8 mm from the bregma to evaluate different regions of the PVN of the hypothalamus. Free‐floating immunofluorescence was performed on all sections evaluated. Briefly, sections were transferred to a 24‐wells plate (1 section/well), and gelatin was removed by incubating the sections for 1 h at 37°C. Sections were then permeabilized in 0.1 M TBS and 0.3% Triton X‐100 for 3 × 15 min in a shaker at room temperature and washed in TBS‐T. Unspecific binding was blocked with 1% BSA, 0.3% Triton x‐100, and 3% normal goat serum in 0.1 M TBS, for 2 h at 24°C. Sections were then incubated with primary antibody for TH (1:1000; Abcam, Cambridge, UK) overnight at 4°C. A negative control was performed by avoiding incubation with primary antibody and keeping in blocking solution for this period. The solution was decanted, and the sections washed in TBS‐T before incubation with specific secondary antibody at a dilution of 1:2000 for 1 h at 24°C in the dark (anti‐mouse Alexa Fluor 488; from Abcam, Cambridge, UK). Slices were washed in TBS in the dark and mounted with a coverslip and a drop of mounting medium Fluoroshield—Sigma‐Aldrich. Each slide was immediately visualized in a Zeiss LSM980 confocal microscope, and fluorescence images were used to measure the fluorescence intensity of TH in the PVN area.

### 2.12 Statistical Analysis

Data were analyzed using GraphPad Prism software, version 9 (GraphPad Software Inc., San Diego, CA, USA) and presented as means with SEM. The significance of the differences between the means was calculated by one‐ and two‐way ANOVA with Bonferroni multiple comparison test. Differences were considered significant at *p* < 0.05.

## 3 Results

### 3.1 CSN Resection Decreases Weight Gain and Improves Glucose Homeostasis

**FIGURE 1:**
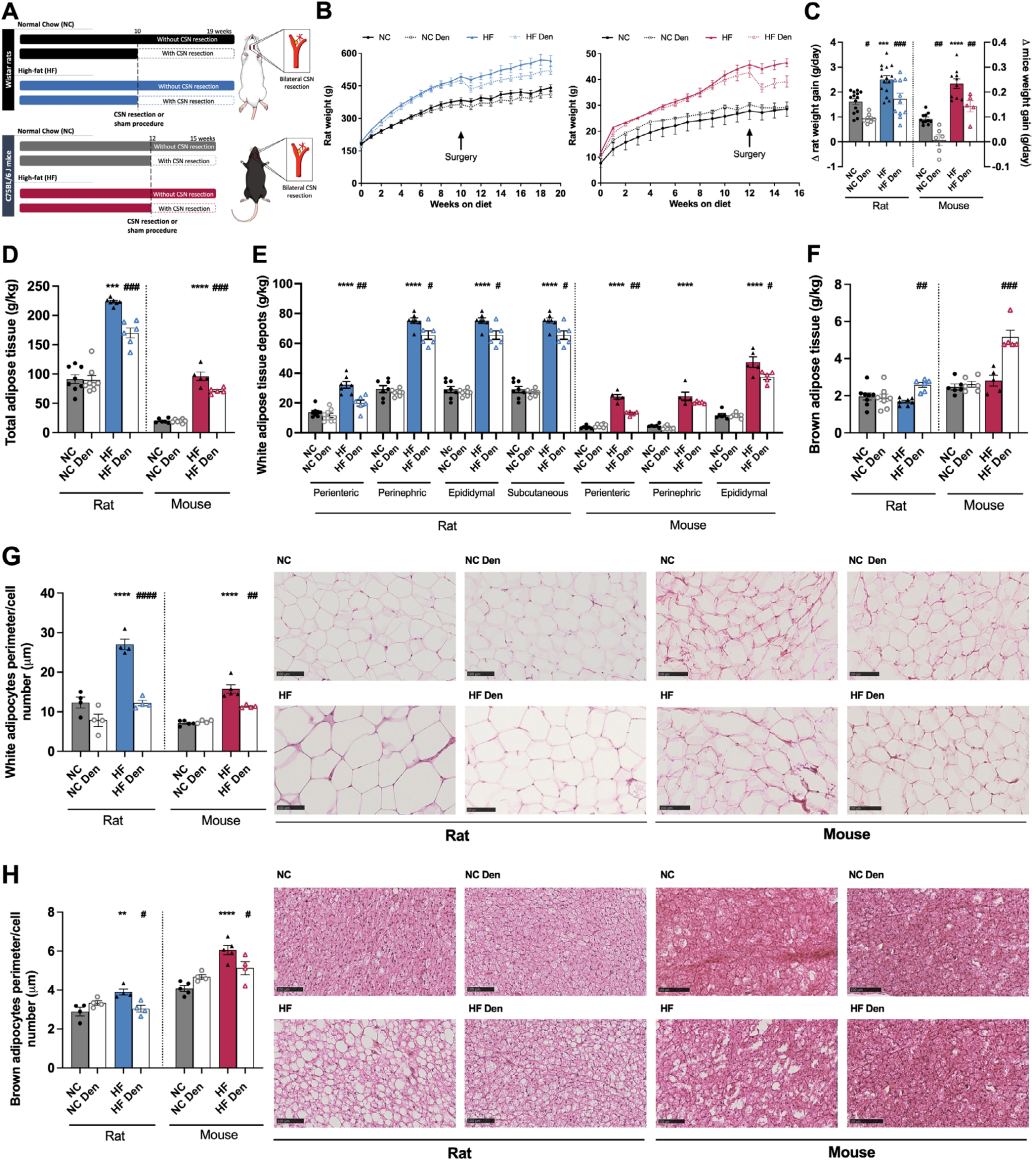
Carotid sinus nerve (CSN) resection/denervation decreases weight gain and adipose tissue deposition in obese dysmetabolic rodents. (A) Schematic representation of the protocol followed throughout the study for rats and mice. Effect of high fat (HF) diet and of CSN resection (CSN Den) on: (B) growth curves of Wistar rats (*n* = 9–15, corresponding to two cohorts of animals that were used for different sets of experiments) (left panel) and C75BL/6J mice (*n* = 5–11) (right panel); (C) average weight gain (g/day) in rats and mice after surgery; (D) total white adipose tissue (WAT) weight (g/kg) (*n* = 5–8); (E) weight (g/kg) of white adipose tissue (WAT) depots in rats and mice (*n* = 5–8); (F) weight (g/kg) of brown adipose tissue (BAT) in rats and mice (*n* = 5–8); (G) visceral WAT adipocytes perimeter per cell number (100 μm)—left panel shows average adipocytes perimeter, right panel shows representative H&E histological images of visceral fat in rat and mice (*n* = 4–5). (H) Brown adipocytes perimeter per cell number (100 μm) in the BAT depot in rats and mice (*n* = 4–5)—left panel shows average adipocytes perimeter, right panel representative H&E histological images of BAT in rat and mice (*n* = 4–5). Black and blue colors represent respectively, normal chow (NC) and HF diet rats. Gray and red colors show respectively NC and HF mice. Den—means animals submitted to CSN denervation/resection. Bars represent mean values ± SEM. Two‐Way ANOVA with Bonferroni multicomparison test. **p < 0.01, ***p < 0.001 and ****p < 0.0001 comparing NC vs. HF groups; #*p* < 0.05, ##*p* < 0.01, ###*p* < 0.001 and ####*p* < 0.0001 comparing values with and without CSN resection.

Diabesity results from a complex interplay between genetic predisposition and environmental factors, with diet playing a crucial role in their development and progression [20]. In line with this, submitting rodents to hypercaloric diets promotes alterations in body weight, SNS activity, blood pressure, and glucose metabolism that are very similar to the human condition [20, 21]. Rats and mice that were fed a lipid‐rich diet (HF diet) during 10 and 12 weeks, respectively (Figure 1A), exhibit a higher growth curve (Figure 1B left and right panels, respectively), increased weight gain (Figure 1C) and total fat amount (Figure 1D) than the animals that were fed a standard diet (normal chow, NC). The increase in fat amount was accompanied by an increase in all the WAT depots studied (Figure 1E). Furthermore, as previously demonstrated [22], HF diet intake promoted a dysmetabolic state, characterized by insulin resistance, glucose intolerance, dyslipidemia, hyperinsulinemia, and increased c‐peptide levels in rats (Table 1), with some dysmetabolic features herein described to be present also in mice (Table 1). Additionally, as previously described by Ribeiro et al. [2], dysmetabolic rats exhibited increased respiratory responses to hypoxia (Figure S1) without alterations in their responses to hypercapnia. These findings, along with the findings that bilateral chronic resection of the CSN—confirmed by the abolition of responses to hypoxia (Figure S1)—decreases weight gain and improves glucose metabolism and insulin action in high‐fat diet (HF) animals of both species (Figure 1C and Table 1) support a role of the CB in the development of diabesity. The effect on weight was clear when the weight gain after surgery was plotted starting 1 week after surgery (Figure 1C). CSN resection decreased weight gain in both NC and HF diet animals. It is noteworthy that obese rats and mice submitted to HF diet and CSN resection decreased the weight gain by 31% and 39%, respectively, compared with HF sham animals (Figure 1C). This decrease in weight gain of HF animals was accompanied by a reduction in the total amount of fat (24% for rats and 26% for mice) (Figure 1D) and by a decrease in all WAT depots (Figure 1E). CSN resection also reverses dysmetabolism, with an improvement of glucose tolerance and a reversion of insulin resistance (Table 1) in rats, as previously described [4, 5], but also in mice. Moreover, CSN resection reversed hyperinsulinemia and dyslipidemia in rats (Table 1). This indicates that the beneficial effects of CSN on metabolism are not species‐specific. Concomitant with the increase in total WAT quantity and distribution, both rats and mice presented increased adipocyte perimeter in their perienteric adipose tissue (Figure 1G), effects that were decreased by CSN resection in rats (55%) and mice (28%), suggesting that the amelioration of metabolism in HF animals induced by CSN resection includes a restructuring of the WAT. Additionally, the HF diet also promoted alterations in the BAT in rats and mice, as evidenced by an increase in adipocyte perimeter (Figure 1H). Interestingly, CSN resection increased the mass of BAT in rats (53%) and mice (84%) (Figure 1F), contributing to a decrease in adipocyte perimeter of 22% and 15%, respectively (Figure 1H). This suggests that the amelioration of metabolism in HF animals induced by CSN resection might include increased energy expenditure.

**TABLE 1:** Effect of carotid sinus nerve (CSN) resection on insulin sensitivity, glucose metabolism, and lipid profile on obese dysmetabolic rats and mice.

Rats					
			Before diet	Before surgery	9 weeks after surgery
Caloric intake (Kcal/day/kg)	NC	Sham	—	197.63 ± 9.68	218.77 ± 30.21
		Denervated	—	215.15 ± 7.14	217.22 ± 19.91
	HF	Sham	—	280.08 ± 29.76*	301.63 ± 17.18*
		Denervated	—	274.63 ± 25.69*	248.45 ± 21.34
Glycemia (mg/dL)	NC	Sham	95.22 ± 5.27	75.11 ± 3.48	82.90 ± 3.60
		Denervated	85.66 ± 3.77	84.44 ± 4.38	85.22 ± 3.71
	HF	Sham	78.22 ± 3.16	95.11 ± 5.06*	92.90 ± 2.20^§§^
		Denervated	81.56 ± 2.12	98.78 ± 4.10**	81.5 ± 2.46^##^
Insulin sensitivity Kitt (%glucose/min)	NC	Sham	4.43 ± 0.24	4.24 ± 0.24	4.78 ± 0.31
		Denervated	5.03 ± 0.33	4.50 ± 0.25	4.85 ± 0.31
	HF	Sham	4.1 ± 0.21	1.76 ± 0.33***^,####^	1.84 ± 0.23^§§§,####^
		Denervated	4.7 ± 0.35	1.92 ± 0.38***	4.24 ± 0.20^###^
Glucose tolerance AUC OGTT (mg/dL × min)	NC	Sham	20 747.55 ± 692.56	18 439.55 ± 810.08	19 966.89 ± 449.27
		Denervated	19 286 ± 498.99	18 160.22 ± 770.48	20 813.45 ± 662.14
	HF	Sham	21 028.67 ± 759.24	25 359.45 ± 825.60***^,##^	24 504.63 ± 355.01^§§^
		Denervated	20 116.11 ± 746.74	25 415.33 ± 689.33***^,####^	23 264.29 ± 433.77^#^
Insulin (pmol/L)	NC	Sham	5.24 ± 1.34	170.39 ± 9.20	240.55 ± 16.00
		Denervated	19.43 ± 2.06	146.68 ± 15.43	227.78 ± 17.16
	HF	Sham	22.98 ± 4.06	415.58 ± 35.34****^,####^	584.21 ± 45.95^§§§§,####^
		Denervated	25.87 ± 1.79	506.94 ± 22.48****^,####^	420.92 ± 21.69^§§§§,####^
C‐peptide (nmol/L)	NC	Sham	0.33 ± 0.09	0.68 ± 0.16	0.64 ± 0.08
		Denervated	0.45 ± 0.12	0.72 ± 0.08	0.76 ± 0.06
	HF	Sham	0.33 ± 0.06	1.34 ± 0.07****^,####^	1.83 ± 0.17^§§§§,###^
		Denervated	0.37 ± 0.10	1.51 ± 0.16****^,####^	1.34 ± 0.15^#^
Cholesterol (mg/dL)	NC	Sham	—	—	61.20 ± 4.47
		Denervated	—	—	63.84 ± 1.54
	HF	Sham	—	—	95.65 ± 4.56****
		Denervated	—	—	77.33 ± 4.14^#^
Triglycerides (mg/dL)	NC	Sham	—	—	90.65 ± 7.02
		Denervated	—	—	91.96 ± 8.21
	HF	Sham	—	—	147.05 ± 24.07*
		Denervated	—	—	108.12 ± 11.06
c‐LDL (mg/dL)	NC	Sham	—	—	4.08 ± 0.31
		Denervated	—	—	4.32 ± 0.17
	HF	Sham	—	—	7.97 ± 1.06***
		Denervated	—	—	5.87 ± 0.54
NEFA (mg/dL)	NC	Sham	—	—	1.06 ± 0.08
		Denervated	—	—	1.10 ± 0.07
	HF	Sham	—	—	1.19 ± 0.07
		Denervated	—	—	0.70 ± 0.18^#^

Mice					
			Before diet	Before surgery	9 weeks after surgery
Caloric intake (Kcal/day/kg)	NC	Sham	—	353.00 ± 12.79	365.70 ± 11.90
		Denervated	—	363.20 ± 14.78	388.20 ± 13.73
	HF	Sham	—	526.30 ± 46.82**	431.40 ± 57.42
		Denervated	—	490.50 ± 43.78*	457.70 ± 40.35
Glycemia (mg/dL)	NC	Sham	—	80.60 ± 2.42	86.40 ± 2.79
		Denervated	—	79.00 ± 3.86	80.33 ± 1.54
	HF	Sham	—	124.50 ± 5.38***	144.75 ± 10.06
		Denervated	—	112.40 ± 7.75**	94.75 ± 11.84^§^
Insulin sensitivity AUC ITT (mg/dL × min)	NC	Sham	—	10 641.20 ± 864.94	9276.60 ± 488.69
		Denervated	—	11 135.83 ± 584.78	10 477.50 ± 1247.50
	HF	Sham	—	21 645.75 ± 1684.90***	23 392.00 ± 695.97
		Denervated	—	22 336.20 ± 2069.03****	15 251.80 ± 1997.12^#,§^
Glucose tolerance AUC OGTT (mg/dL × min)	NC	Sham	—	23 194.80 ± 678.63	19 657.60 ± 971.99
		Denervated	—	20 851.33 ± 2783.03	18 667.83 ± 535.42
	HF	Sham	—	32 773.25 ± 1155.82***	30 596.35 ± 1111.42
		Denervated	—	33 064.60 ± 2783.03**	26 894.00 ± 1191.86

*Note:* Values represent means ± SEM; Two‐Way ANOVA with Bonferroni multicomparison test. *p < 0.05, **p < 0.01, ***p < 0.001 and ****p < 0.0001 comparing normal chow animals and HF values; ^#^ *p* < 0.05, ^##^ *p* < 0.01, ^###^ *p* < 0.001 and ^####^ *p* < 0.0001 comparing values without and with CSN resection; ^§^ *p* < 0.05, ^§§^ *p* < 0.01, ^§§§^ *p* < 0.001, ^§§§§^ *p* < 0.0001 comparing values 9 weeks after CSN with values 9 weeks after sham. Glucose tolerance measured as AUC glucose in OGTT (mg/dL × min). Insulin tolerance measured as Kitt (%glucose/min) in rats and as AUC glucose in ITT (mg/dL × min) in mice.

Abbreviations: AUC, area under the curve; c‐LDL, low density lipoproteins cholesterol; HF, high fat; Kitt, constant of the insulin tolerance test; NEFA, non‐esterified free fatty acids; OGTT, oral glucose tolerance test.

### 3.2 CSN Resection Enhances Visceral WAT and BAT Function by Increasing Their Metabolism

**FIGURE 2:**
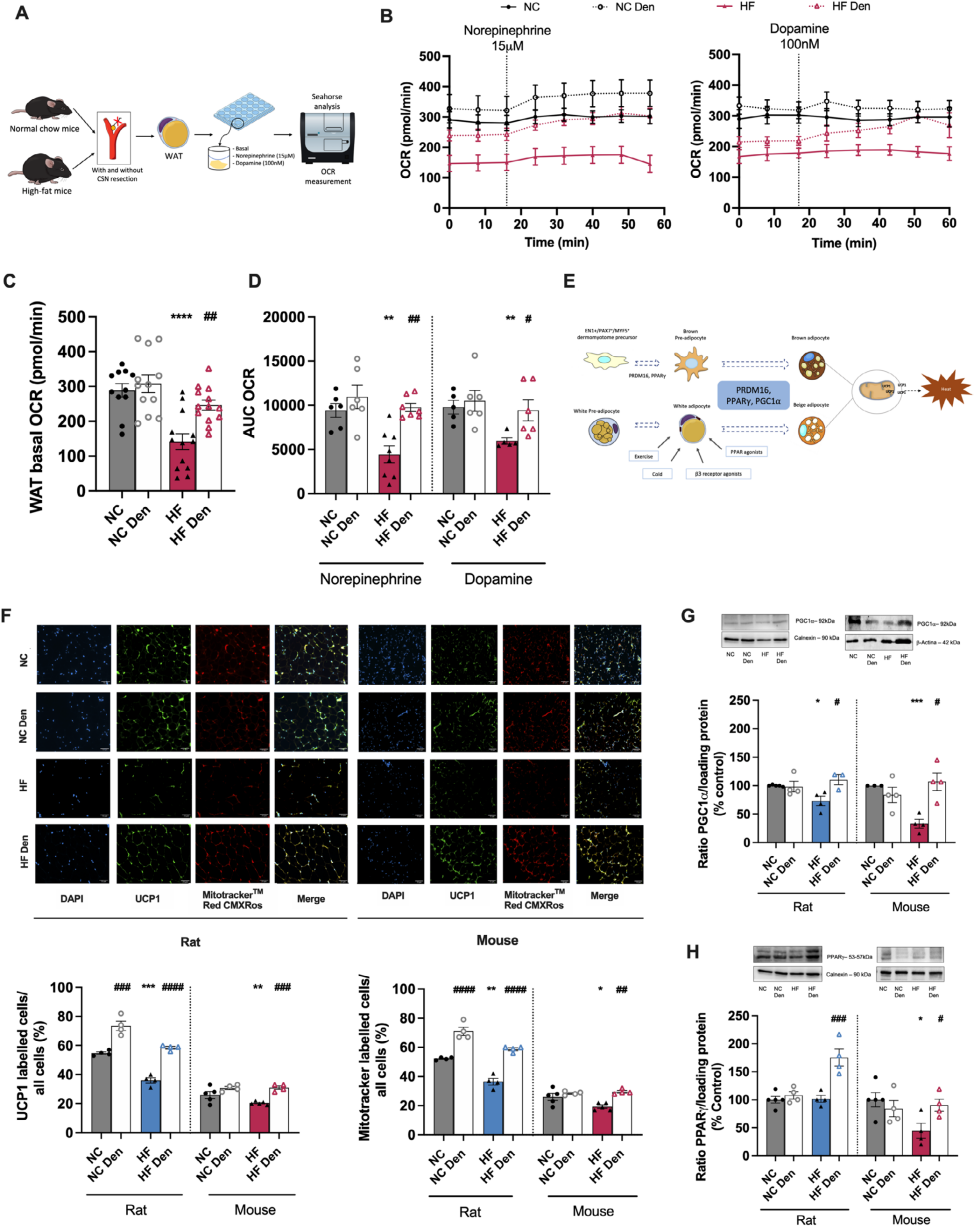
Ablation of carotid body (CB) activity through carotid sinus nerve (CSN) resection heightens visceral white adipose tissue (WAT) metabolism in obese dysmetabolic rodents. (A) Panel shows a schematic illustration of the protocol used to evaluate mice WAT oxygen consumption rate (OCR). From (B) to (H) is described the effect of high fat (HF) diet and of CSN resection on the: (B) OCR per minute, reflecting adipose tissue metabolism, before and after stimulation with norepinephrine [15 μM] (left panel) or dopamine [100 nM] (right panel) in mice (3 pieces of tissue from 4–6 animals); (C) average basal OCR in mice (15–27 pieces of tissue from 4–6 animals) before the stimulation with norepinephrine and dopamine; (D) average OCR after stimulation with norepinephrine [15 μM] or dopamine [100 nM] (3 pieces of tissue from 4–6 animals); (E) Illustration of the molecular markers involved in brown adipocytes differentiation as well as the stimuli involved in the beiging of WAT; (F) average expression of PGC1α (92 kDa) (*n* = 3–5); (G) average expression of PPARγ (53‐57 kDa) on visceral WAT of rats and mice (*n* = 4–5)—representative western blots are shown on the top of the graphs; and (H) percentage of UCP1 protein labeled cells and percentage of mitotrackerTM Red CMXRos labeled cells (top panels) in the perienteric depot (*n* = 4–5) in rats and mice; right panels show representative images of UCP1 and MitotrackerTM Red CMXRos labeled cells, Green—UCP1 labeled adipocytes; Red—MitotrackerTM Red CMXRos labeled adipocytes; Blue—DAPI labeled nuclei of the adipocytes; Yellow—Merge of UCP1 and MitotrackerTM Red CMXRos labeled cells. Gray and blue colors represent, respectively, normal chow (NC) and HF rats. Gray and red colors show NC and HF mice, respectively. Den—means animals submitted to CSN denervation/resection. Bars represent mean values ± SEM. Two‐Way ANOVA with Bonferroni multicomparison test. *p < 0.05, **p < 0.01, ***p < 0.001 and ****p < 0.0001 comparing NC vs. HF groups, ^#^ *p* < 0.05, ^##^ *p* < 0.01, ^###^ *p* < 0.001 and ^###^ *p* < 0.0001 comparing values with and without CSN resection.

**FIGURE 3:**
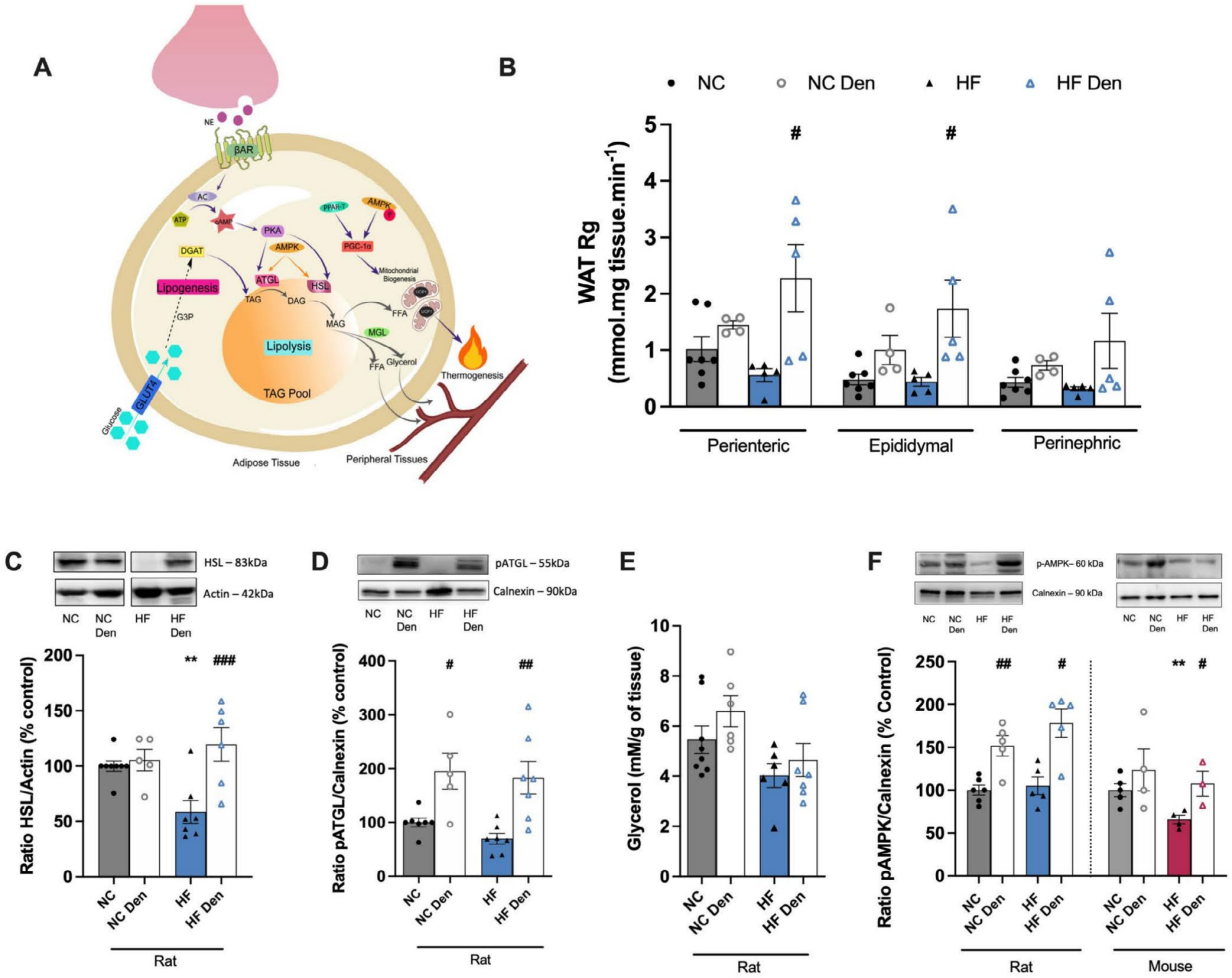
Carotid body (CB) modulates lipid fluxes in white adipose tissue (WAT) in rodents. (A) Illustration of the molecular markers involved in adipose tissue energy expenditure. From (B) to (F) is described the effect of high fat (HF) diet and of CSN resection on: (B) Rg′ values, reflecting glucose uptake on WAT depots in rats (*n* = 4–7); (C) WAT average expression of HSL (83 kDa) on WAT of rats (*n* = 5–8); (D) WAT average expression of phosphorylated ATGL (pATGL) (55 kDa) in rats (*n* = 5–7)—representative western blots are shown on the top of the graphs; (E) glycerol levels, as an index of lipolysis (*n* = 6–8) in WAT in rats; (F) WAT average expression of phosphorylated AMPK (pAMPK) (60 kDa) in rats and mice (*n* = 3–6)—representative western blots are shown on the top of the graphs. Gray and blue colors represent, respectively, normal chow (NC) and HF diet rats. Gray and red colors show NC and HF mice, respectively. Den—means animals submitted to CSN denervation/resection. Bars represent mean values ± SEM. Two‐Way ANOVA with Bonferroni multicomparison test. **p < 0.01 comparing NC vs. HF groups; ^#^ *p* < 0.05, ^##^ *p* < 0.01 and ^###^ *p* < 0.001 comparing values with and without CSN resection.

We assessed visceral WAT metabolism by measuring basal oxygen consumption rate (OCR) and sympathetic‐evoked OCR (NE [15 μM] or dopamine [100 nM]) using Seahorse technology in mice (Figure 2A,B, left and right panels). It is well established that mitochondrial morphology, mass, and function are impaired in multiple adipose tissue depots in obese rodents [23]. In accordance with the aforementioned observations, we observed that the visceral WAT basal OCR of mice submitted to an HF diet decreased by 51% in comparison with NC animals (Figure 2C). Interestingly, CSN resection in HF animals restored WAT OCR by promoting an increase of 77% (Figure 2C). It has been demonstrated that sympathetic mediators, such as NE [24, 25] or dopamine [26, 27] activate WAT. Consequently, we tested NE and dopamine on OCR and observed that NE and dopamine‐evoked OCR were reduced by 53% and 44% in WAT of HF mice in comparison with NC animals. CSN resection was found to increase WAT responses to NE and dopamine in HF animals (55% in response to NE and 77% in response to dopamine) (Figure 2D). To confirm a possible increase in adipose tissue energy expenditure mediated by CSN resection on dysmetabolic states, we evaluated mitochondrial density and UCP1 immunolabeling (Figure 2F) in the visceral WAT. The HF diet was found to decrease UCP1 levels (Figure 2F, left panel and left bottom graph) and mitochondrial density (Figure 2F, right panel and right bottom graph) in perienteric adipose tissue depot in rats and mice. Bilateral CSN resection resulted in increased UCP1 immunolabeling and mitochondrial density in both species. In dysmetabolic rats and mice, UCP1 levels increased by 39% and 52%, respectively, while mitochondrial density increased by 61% and 52%, respectively. These findings suggest a restoration of WAT metabolism through increased thermogenesis (Figure 2F). To confirm this phenotype, we assessed the levels of peroxisome proliferator‐activated receptor gamma coactivator 1 alpha (PGC1α) and peroxisome proliferator‐activated receptor gamma (PPARγ) proteins, which are key mediators of lipid metabolism and markers of brown fat phenotype respectively (Figure 2E). The HF diet results in a 27% and 67% reduction in the PGC1α levels in rats and mice respectively, which was completely reversed by CSN resection (Figure 2G). Interestingly, the HF diet did not alter the expression of PPARγ in visceral WAT of rats while decreasing it by 55% in the WAT of mice. CSN resection increased PPARγ expression by 73% in the HF rats and completely restored its levels in mice to the levels observed in the control group (Figure 2H). Glucose uptake has been widely used as a surrogate marker for thermogenesis and energy balance since glucose is one of the substrates for adipocyte metabolism [26]. Here, we evaluated glucose uptake, in vivo, in WAT depots, and the levels of hormone‐sensitive lipase (HSL) as well as the phosphorylated levels of adipose triglyceride lipase (ATGL) and AMP‐activated protein kinase (AMPK) protein levels by western blot in rats (Figure 3). The HF diet was found to have a non‐significant decreased glucose uptake in perienteric fat (Figure 3B). In striking contrast, CSN resection was observed to increase glucose uptake in all fat depots in normal chow and HF animals, with a significantly different effect in the perienteric depot of the HF animals (Figure 3B). Moreover, we found that the HF diet resulted in a decrease in HSL levels, without changing pATGL levels. In contrast, CSN resection led to an increase in both HSL and pATGL in both NC and HF animals (Figure 3C,D), while glycerol levels remained unchanged (Figure 3E). This suggests that CSN resection may have increased WAT lipid fluxes to generate FFA for thermogenesis (Figure 3A). Finally, knowing that AMPK regulates HSL and ATGL, we evaluated the effect of HF diet and of CSN resection on its activity, assessed by measuring the levels of its phosphorylated form (Figure 3F). HF diet did not alter phosphorylated AMPK levels in rats; however, we did observe a 34% decrease in obese mice (Figure 3F). CSN resection increased AMPK phosphorylation by 52% and 70% in the CTL and HF rats, respectively, and restored the phosphorylation levels of AMPK in dysmetabolic mice (Figure 3F).

**FIGURE 4:**
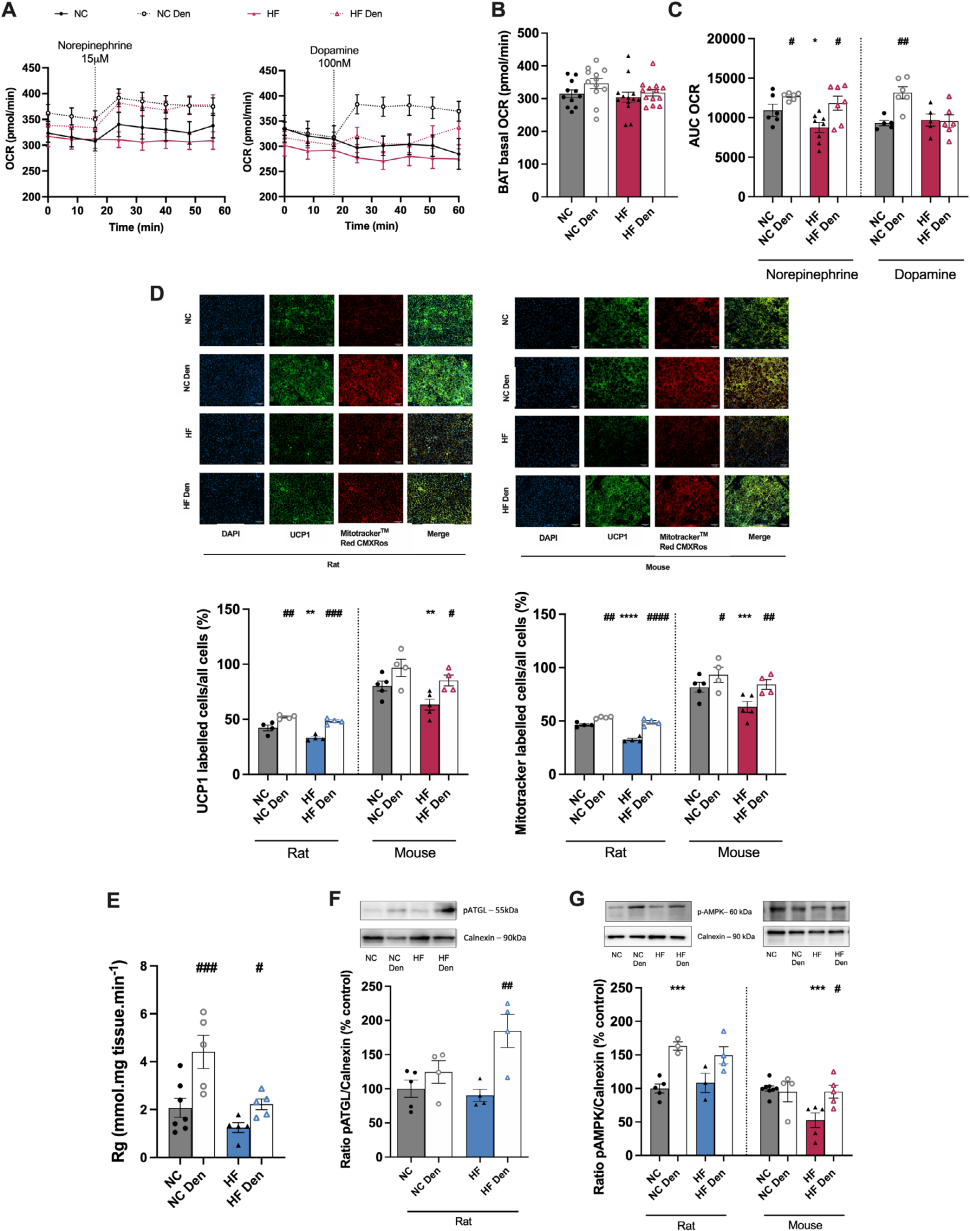
Carotid sinus nerve (CSN) resection improves brown adipose tissue (BAT) metabolism in rodents. Effect of high fat (HF) diet and of CSN resection on: (A) Curves of oxygen consumption rate (OCR) per minute, reflecting adipose tissue metabolism, before and after stimulation with norepinephrine [15 μM] (left panel) or dopamine [100 nM] (right panel) in the BAT of mice (*n* = 18–27 pieces of tissue from 6–8 animals). (B) Average basal OCR in the BAT in mice (*n* = 18–27 pieces of tissue from 6–8 animals) before the stimulation with norepinephrine and dopamine; (C) Average OCR after stimulation with norepinephrine [15 μM] or dopamine [100 nM] (*n* = 5–8 animals); (D) percentage of UCP1 protein labeled cells and percentage of mitotrackerTM Red CMXRos labeled cells (top panels) in BAT (*n* = 4–5) in rats and mice; bottom panels show representative images of UCP1 and MitotrackerTM Red CMXRos labeled cells, Green—UCP1 labeled adipocytes; Red—MitotrackerTM Red CMXRos labeled adipocytes; Blue—DAPI labeled nuclei of the adipocytes; Yellow—Merge of UCP1 and MitotrackerTM Red CMXRos labeled cells; (E) Rg′ values, reflecting glucose uptake on BAT depots in rats (*n* = 5–7); (F) BAT average expression of pATGL (55 kDa) in rats (*n* = 4–5); (G) average expression of pAMPK (60 kDa) on BAT of rats and mice (*n* = 3–8)—representative western blots are shown on the top of the graphs. Gray and blue colors represent, respectively, normal chow (NC) and HF diet rats. Gray and red colors show NC and HF mice, respectively. Den—means animals submitted to CSN denervation/resection. Bars represent mean values ± SEM. Two‐Way ANOVA with Bonferroni multicomparison test. *p < 0.05, **p < 0.01, ***p < 0.001 and ****p < 0.0001 comparing NC vs. HF groups; ^#^ *p* < 0.05, ^##^ *p* < 0.01, ^###^ *p* < 0.001 and ^####^ *p* < 0.0001 comparing values with and without CSN resection.

It is widely accepted that dysmetabolic states are also associated to BAT dysfunction, characterized by the accumulation of enlarged lipid droplets and mitochondrial dysfunction [28]. To study BAT mitochondrial function, we evaluated basal OCR as well as the OCR in response to NE [15 μM] or dopamine [100 nM] in mice (Figure 4A–C). Our findings indicate that neither the HF diet nor CSN resection significantly altered basal OCR (Figure 4A,B). As expected, NE increased OCR in BAT in NC animals (Figure 4A,C), with this effect being decreased by 20% in obese mice (Figure 4C). CSN resection augmented NE activation of BAT by 23% and 42%, respectively, in the NC and obese mice (Figure 4C). Dopamine is described to directly activate thermogenesis and to increase mitochondrial mass in brown adipocytes [29]. In this study, we observed that dopamine did not elicit an increase in OCR both in NC and obese mice (Figure 4A right panel). However, in CSN‐resected animals' dopamine increased OCR by 37% in mice fed with a standard diet, with no changes observed in dysmetabolic mice (Figure 4A–C). In order to ascertain whether there was a correlation between OCR and increased thermogenesis, we evaluated mitochondrial density by immunohistochemistry. The HF diet resulted in a decrease of 21% in UCP1 labeled cells, in both species (Figure 4D, top panel). In agreement, mitochondrial density decreased by 30% and 23%, respectively, in rats and mice submitted to the HF diet (Figure 4D, bottom panel). Bilateral CSN resection increased UCP1 immunostaining and mitochondrial density in NC animals and reversed the impact of the HF diet on these parameters in both species. In agreement with a high metabolic activity, CSN‐resected animals fed with NC and HF diet exhibited an increased BAT glucose uptake, as evidenced by in vivo measurements (Figure 4E). Finally, as observed in visceral WAT, no alterations were found in phosphorylated ATGL (Figure 4F) and AMPK (Figure 4G) in rats following an HF diet. However, a decrease of 48% in AMPK was observed in obese mice. CSN resection resulted in a 104% increase in ATGL phosphorylation (Figure 4F) and a 38% increase in AMPK phosphorylation in rats. Furthermore, it restored AMPK levels in mice, with an 81% increase compared to the HF mice (Figure 4G).

### 3.3 CSN Resection Rescues Adipose Tissue Sympathetic/Catecholamine Resistance in Dysmetabolic States

**FIGURE 5:**
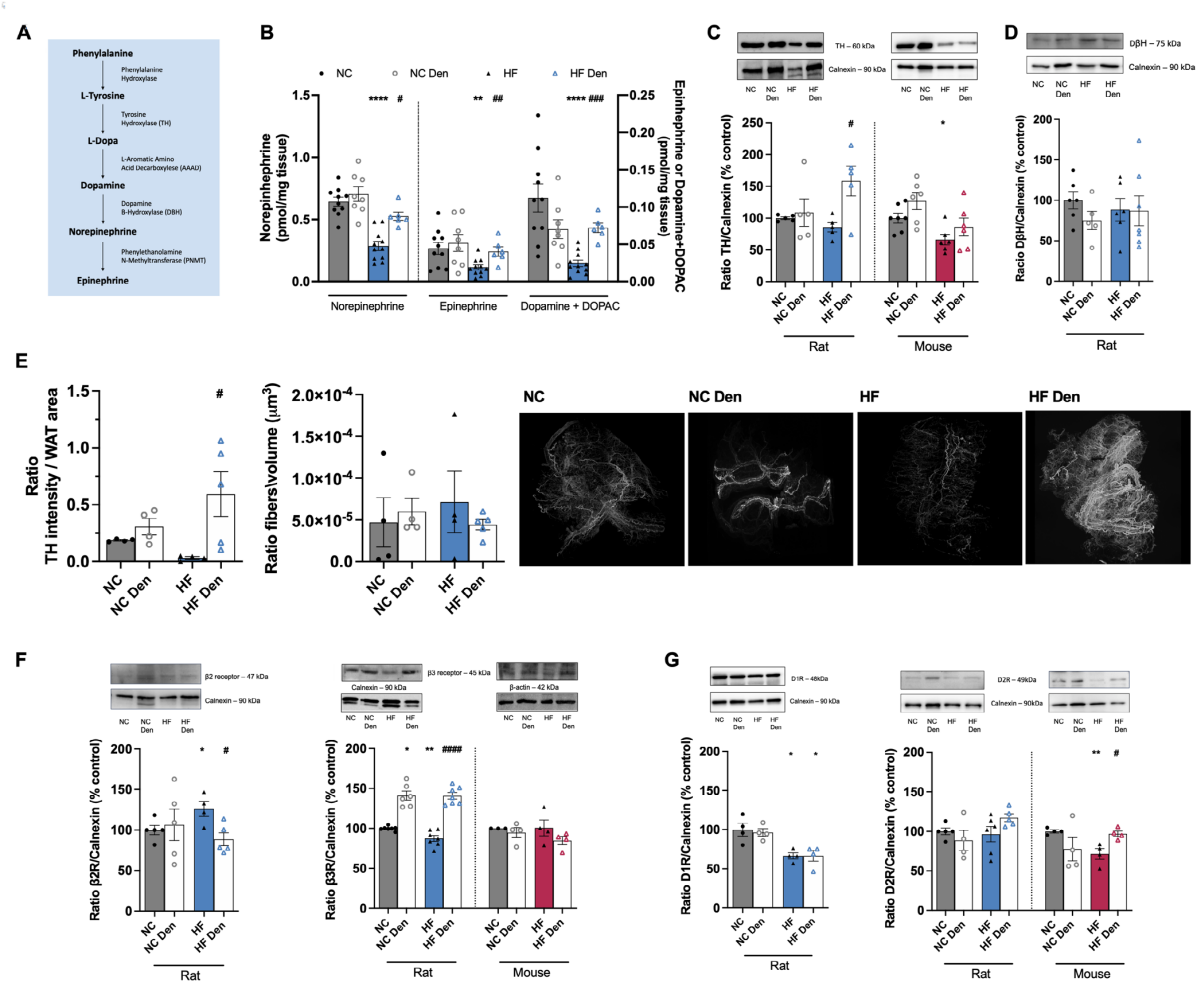
Ablation of carotid body (CB) activity through carotid sinus nerve (CSN) resection restores sympathetic inputs in visceral white adipose tissue (WAT). (A) schematic representation of the catecholamine biosynthesis pathways; (B) effect of HF diet and CSN resection on the levels of dopamine + DOPAC, norepinephrine and epinephrine in the WAT. From (C) to (E)—Sympathetic innervation in the WAT presented by: (C) WAT average tyrosine hydroxylase (TH) levels (60 kDa) in rats and mice (*n* = 5–7); (D) WAT average dopamine β hydroxylase (DβH) levels (75 kDa) in rats; (E) from the left to the right: Intensity and fibers volume of TH immunolabeling (*n* = 4–5) in WAT of rats—representative images are shown at the right panel, for animated gif of the images consult the videos White adipose tissue (WAT); (F) average β2 receptors (β2R, 47 kDa) in rats (left panel) and β3 receptors (β3R, 45 kDa) in rats and mice (right panel) (*n* = 3–7); and (G) average dopamine type 1 receptors (D1R, 48 kDa) in rats (left panel) and dopamine type 2 receptors (D2R, 49 kDa) in rats and mice (left panel) (*n* = 4–6). Representative western blots are shown on the top of the graphs. Gray and blue colors represent, respectively, normal chow (NC) and HF diet rats. Gray and red colors show NC and HF mice, respectively. Bars represent mean values ± SEM. Two‐Way ANOVA with Bonferroni multicomparison test. *p < 0.05, **p < 0.01 and ****p < 0.0001 comparing NC vs. HF groups; ^#^ *p* < 0.05; ^##^ *p* < 0.01; ^###^ *p* < 0.001 and ^####^ *p* < 0.0001 comparing values with and without CSN resection.

Obesity and dysmetabolic states have been linked to a systemic overactivation of the SNS [30]. This is evidenced by our findings that dysmetabolic rats exhibit an overactivation of the SNS, reflected by 118% and 162%, increases in plasma NE and epinephrine, respectively (Table S1). Furthermore, the SNS index, as evaluated by heart rate variability analysis, has been found to be elevated by 46% in these animals, with no changes in the parasympathetic index (Table S1). As expected, and in agreement with the findings that CB activation leads to a whole‐body overactivation of the SNS [12, 31], CSN resection normalized the effects of the HF diet on plasma epinephrine and attenuated NE plasma levels, as well as restoring the SNS index (Table S1). In contrast with the whole‐body overactivation of the SNS present in dysmetabolic states (Table S1, [5, 21, 24]) and in line with literature showing a regional activation of the SNS [32, 33] and that sympathetic activation increase adipose tissue metabolism [34], catecholamines levels within visceral WAT, namely NE, epinephrine and dopamine were decreased by 55%, 50% and 82%, respectively, in HF rats (Figure 5B). These effects were almost completely reversed by CSN resection (Figure 5B). The decreased catecholamines content within the visceral WAT in obese animals was coincident with a 34% reduction in the TH levels in HF mice, as evaluated by Western Blot (Figure 5C). Furthermore, a decrease of 83% in the intensity of TH innervation was measured by light‐sheet microscopy in the HF rats, with no significant alterations in the volume of fibers (Figure 5E, video WAT_NC_SHAM vs. video WAT_HF_SHAM White adipose tissue (WAT)). The establishment of a link between the CB and adipose tissue catecholaminergic signaling was evidenced by 85% and 30% increase in the TH levels following CSN resection in dysmetabolic rats and in mice, respectively (Figure 5C). Additionally, the intensity of TH immunostaining evaluated by light‐sheet microscopy increased by 174% in these animals, without changing the volume of sympathetic fibers innervating visceral WAT (Figure 5E, left panel, videos WAT_NC_DEN and WAT_HF_DEN White adipose tissue (WAT)). No changes in dopamine β‐hydroxylase (DβH) levels were observed in WAT in HF and/or CSN‐resected animals (Figure 5D), suggesting that the levels of DβH are enough to maintain the conversion rate of dopamine into NE. Knowing that catecholamine resistance is a key feature of the dysmetabolic states [35, 36] and is associated with altered levels of β‐adrenergic receptors including the downregulation of β3‐adrenergic receptors [36] we evaluated the levels of these receptors. Consistently, we found that HF diet decreased β3 receptors levels by 13% (Figure 5F, right panel) and promoted a decrease of both D1 and D2 receptors by 34% and 29%, respectively in WAT (Figure 5G, left and right panels), while increasing β2 receptors by 26% (Figure 5E, left panel). On the other hand, CSN resection in the WAT decreased β2 receptors expression by 30% while increasing β3 levels by 61% in rats (Figure 5F, left and right panels). Altogether, these results indicate that the CB controls sympathetic innervation to visceral WAT and that CSN resection restores catecholaminergic integration.

**FIGURE 6:**
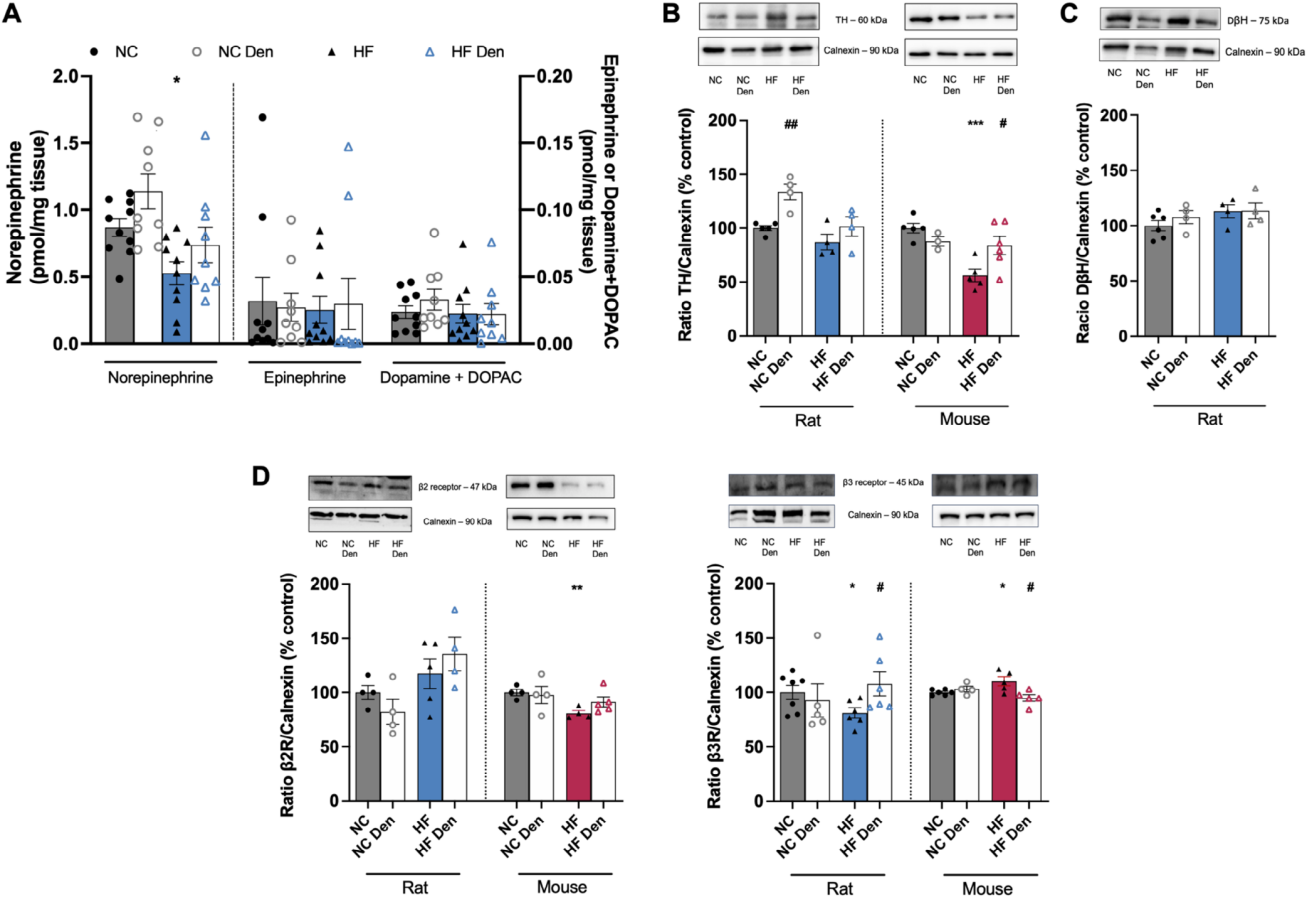
Carotid body (CB) regulation of norepinephrine (NE) action on β3 adrenergic receptor is key to brown adipose tissue (BAT) metabolic function. (A) effect of high fat (HF) diet and carotid sinus nerve (CSN) resection on the levels of dopamine + DOPAC, NE and epinephrine in the BAT. From (B) to (D): Sympathetic innervation to BAT assessed as: (B) BAT average TH expression (60 kDa) in rats and mice (*n* = 4–6); (C) BAT average D𝛃H levels (60 kDa) in rats and mice (*n* = 4–6); (D) BAT average 𝛃2 receptors (𝛃2R, 47 kDa) in rats (left panel) and 𝛃3 receptors (𝛃3R, 45 kDa) in rats and mice (right panel) (*n* = 3–7); Representative western blots are shown on the top of the graphs. Gray and blue colors represent, respectively, normal chow (NC) and HF diet rats. Gray and red colors show NC and HF mice, respectively. Den—means animals submitted to CSN denervation/resection. Bars represent mean values ± SEM. Two‐Way ANOVA with Bonferroni multicomparison test. *p < 0.05; **p < 0.01 and ***p < 0.001 comparing NC vs. HF groups; ^#^ *p* < 0.05 and ^##^ *p* < 0.01 comparing values with and without CSN resection.

In line with a major role for NE in BAT modulation and induced‐thermogenesis [37], only NE levels were found to be decreased in HF rats (Figure 6A). This may reflect the observed decrease in TH levels in HF diet animals, which was significant in mice (Figure 6B), although with no change in DβH levels (Figure 6C). CSN resection in HF animals restored BAT TH levels to control levels in mice and increased TH levels in rats (Figure 6B). Moreover, it attenuated the decrease in NE levels within the tissue (Figure 6A). Interestingly, the HF diet did not alter β2 receptor levels in rats but reduced β3 levels by 19%. Conversely, in mice, the HF diet decreased β2 expression while increasing β3 expression by 19% and 10%, respectively (Figure 6D, left and right panels), suggesting distinct regulatory mechanisms and contributions of β receptors in the BAT of mice and rats. CSN resection restored β3 receptor levels in rats and mice (Figure 6D), suggesting that CB regulation of NE action on β3 receptor is key to BAT metabolic function.

### 3.4 Carotid Sinus Nerve Enhances Tyrosine Hydroxylase (TH)‐Positive Labeling at the Paraventricular Nucleus (PVN) of the Hypothalamus

**FIGURE 7:**
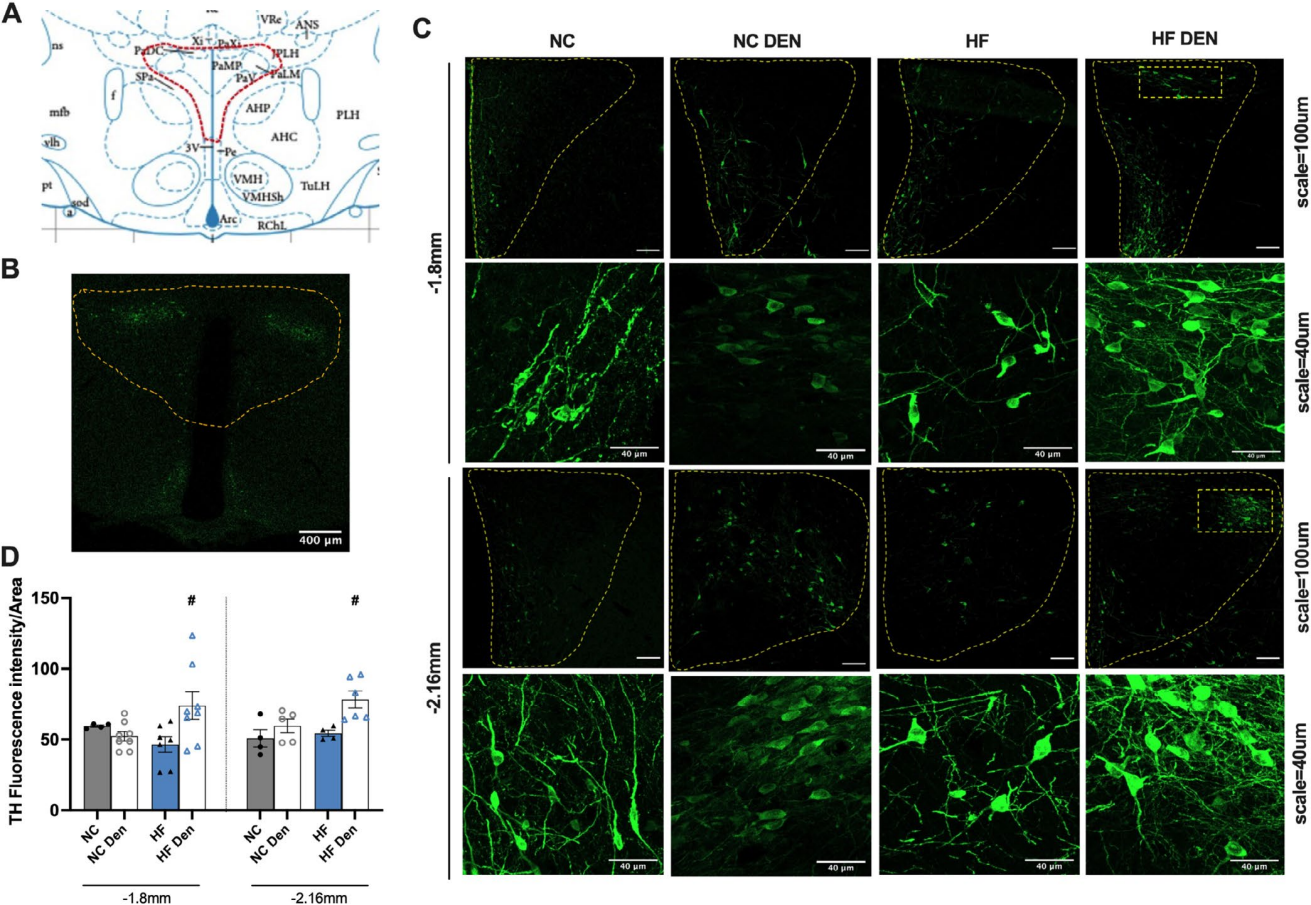
Ablation of carotid activity via carotid sinus nerve (CSN) resection heightens tyrosine hydroxylase (TH)‐positive labeling at paraventricular nucleus (PVN) of the hypothalamic in obese dysmetabolic animals. (A) Illustration of the PVN region analyzed for TH immunostaining obtained in the Paxinos & Watson Atlas for rat brain; (B) Representative image of TH immunostaining in a brain slice of a normal chow rat where the PVN region is delimited in yellow. Effect of high fat (HF) diet and of CSN resection on the: (C) Representative images of TH‐positive labeling (green labeling) with lower (scale = 100 μm) and higher magnification (scale = 40 μm) in the PVN, at −1.8 mm and −2.16 mm coordinates of the bregma, for rats. (D) Mean TH‐positive immunostaining in the PVN coordinates −1.8 mm and −2.16 mm from the bregma. Gray and blue colors represent, respectively, normal chow (NC) and HF diet rats. Bars represent mean values ± SEM. Two‐Way ANOVA with Bonferroni multicomparison test. ^#^ *p* < 0.05 comparing values with and without CSN resection.

Knowing that CB inputs are integrated in the PVN [16], one of the most important autonomic control nuclei in the brain regulating metabolism and energy balance [17] and that TH‐positive neurons regulate glucose homeostasis [18], we therefore tested whether CB inputs to WAT are controlled by TH‐positive neurons within the PVN (Figure 7). We observed that HF diet promotes a tendency to decrease TH‐positive labeling by 20.7% at −1.8 mm coordinates in the PVN with no changes at −2.16 mm coordinates (Figure 7C,D). Additionally, CSN resection increased TH‐positive labeling at both coordinates of the PVN to levels above those in NC animals and by 65.2% and 43.5% in the −1.8 mm and −2.16 mm coordinates, respectively, in comparison with HF diet animals (Figure 7D). Interestingly, the TH‐positive neuronal subpopulations activated in CSN‐resected animals appear to be located in the lateral parvocellular region of the PVN (both at −1.8 mm and −2.16 mm coordinates), known to be involved in the regulation of the autonomic nervous system [38], and in the periventricular subnuclear region (−2.16 mm coordinates).

## 4 Discussion

Herein, we described for the first time a unique mechanism by which the CB controls WAT and BAT metabolism through restoration of sympathetic drive to the adipose tissue in favor of glucose and energy homeostasis. Such functional plasticity is induced by resection of the CSN, and thus CB ablation, and leads to an improvement in metabolism characterized by decreased weight gain and an amelioration of glucose homeostasis, underscoring the critical role of the CB in the development and progression of dysmetabolic states. Importantly, CSN resection enhanced TH‐positive labeling in the PVN of the hypothalamus of dysmetabolic animals, suggesting not only a modulation of sympathetic drive to the adipose tissue but also TH levels within the tissue, impacting dopaminergic and noradrenergic levels. Consequently, this process normalizes baseline visceral WAT metabolism and improves sympathetic activation of BAT. Therefore, the CB is a key player in the hypothalamus–SNS–adipose tissue circuit activity controlling glucose and energy homeostasis. Importantly, these mechanisms are well conserved in rodents.

Consistent with the known deleterious effects of hypercaloric diets [39, 40], Wistar rats and C57BL/6 mice submitted to 19 or 15 weeks of a 60% lipid‐rich diet, respectively, exhibited a phenotype consistent with that observed in obese humans: (1) marked increase in weight gain accompanied by increased fat deposition, coincident with an increase in adipocyte perimeter in WAT and BAT; (2) development of dysmetabolism characterized by insulin resistance, glucose intolerance, hyperinsulinemia, reduction in phosphorylated AMPK expression, and dyslipidemia. As expected, the HF diet contributed to a decrease in WAT and BAT metabolism, characterized by decreased OCR in WAT in basal conditions and in response to the catecholaminergic mediators NE and dopamine, as well as to a decrease in thermogenesis markers and a reduction in the levels of PGC1α and PPARγ, critical regulators of mitochondrial biogenesis. In agreement with the increased weight gain and body fat mass in HF rodents, the increase in WAT depots was accompanied by adipocyte hypertrophy due to increased nutrient accumulation, as described in the literature in rodents [39, 40, 41] and in humans [42]. The HF diet also decreased BAT volume and contributed to increased lipid deposition within this tissue, with large lipid droplets accumulating within brown adipocytes with increased adipocyte size. By contrast, some studies report an increase in BAT weight [43, 44] when using C57BL/6 mice and with different durations of exposure to the HF diet. Nevertheless, the increase in adipocyte size and the accumulation of lipid droplets within the brown adipocytes observed here are consistent with the findings of others [43, 44]. These different results might also be explained by differences in tissue collection and processing (in the present study the WAT around the BAT depots was dissected and weighed separately from the BAT depot).

WAT exhibited lower oxygen consumption rates than the BAT in lean animals, which is in line with the idea that adipose tissue oxygen consumption is relatively low in lean healthy subjects, accounting for approximately 5% of whole‐body oxygen consumption [45]. As expected, the HF diet decreased basal WAT OCR, and reduced NE and dopamine‐evoked OCR, suggesting that the HF diet is not only affects adipocyte mitochondrial activity but also affects adipocyte responses to SNS stimuli and consequently lipolysis [44]. This is consistent with the observed decrease levels in HSL and pAGTL, which may reflect an accumulation of nutrients within the adipocyte and therefore an increase in adipocyte size. These results on the effects of HF diet on WAT metabolism are in accordance with the general idea that HF diet leads to a decreased WAT metabolism. Accordingly, we found that UCP1 and Mitotracker immunolabeling, in addition to PGC1α and PPARγ proteins, were decreased in HF animals. In accordance with the decreased basal OCR activity in WAT induced by the HF diet, we also observed that HF diet decreased PGC1α, PPARγ and phosphorylated AMPK in WAT. In contrast, the HF diet did not alter either basal BAT OCR or dopamine‐stimulated OCR in obese mice but decreased the NE‐evoked OCR in the BAT as well as UCP1 protein expression and mitochondrial density. In accordance with the decreased basal OCR activity in WAT and no effect on BAT induced by the HF diet, we also observed that HF diet decreased PGC1α, PPARγ and phosphorylated AMPK in WAT, with no alterations in the BAT. Consistent with our results, other studies suggest that in obese models, PPARγ levels are decreased in AT [45] and PGC1α mRNA levels are decreased not only in the WAT [46] and BAT [47] but also in skeletal muscle [48].

Obesity and dysmetabolism are often associated with a generalized whole‐body SNS overactivity, particularly in renal outflow [49], with obese adults presenting increased urinary and plasma NA levels [50], that extend to the skeletal muscle vasculature [51]. Moreover, animal [52] and clinical studies [24] have demonstrated that increased SNS activity is involved in the pathophysiology of altered cardiac structure and function in essential hypertension, and that obese insulin‐resistant individuals display have blunted sympathetic neuronal responses to physiological hyperinsulinemia, glucose consumption and changes in energy status [53]. In agreement with these studies and as shown by our group in animal models of prediabetes and type 2 diabetes [2, 4], we observed increased plasma levels of the NE and epinephrine and whole‐body SNS index in the obese rats, demonstrating overall SNS overactivity.

The SNS directly innervates both WAT and BAT and plays a key role in modulating lipolysis and/or energy expenditure/thermogenesis through the release of NE and dopamine [14] and the activation of their respective receptors [13, 54]. For example, β3 agonists have been shown to induce thermogenesis in the WAT, and BAT [55, 56, 57] and dopamine has been shown to directly increase mitochondrial mass and thermogenesis in the BAT [29]. More recently, dopamine has also been shown to potentiate the effect of insulin on glucose uptake in visceral WAT and to promote adipose tissue metabolism [26]. Here, we show that the hypercaloric dietary intake produced a decrease in basal metabolism in WAT, as assessed by measurement of OCR, as well as a decrease in mitochondrial activity, UCP1 expression, PPARγ and PGC1α, and decreased catecholaminergic activation in WAT and BAT. These results are in agreement with the decreased levels of catecholamines found in WAT and BAT and with the low intensity of TH expression in the AT measured by light‐sheet microscopy. On the surface, these results appear to contradict the idea that obesity and its metabolic comorbidities are associated with a SNS overactivation. However, recent data suggest that tissue‐specific activation/modulation of the SNS occurs [58, 59]. Accordingly, our data clearly show that the HF diet decreased catecholaminergic activation of the AT while increasing whole‐body SNS activity.

Consistent with this we have shown that CSN resection not only attenuates sympathetic overactivation [5, 60], but also increases sympathetic integration and catecholaminergic action in the adipose tissue in a manner that restores or even increase WAT and BAT metabolism. This consistently reduces weight gain and adipose tissue deposition in obese rodents, contributing to a reduction of adipocyte size and reversal of metabolic dysfunction. Moreover, CSN resection ameliorated visceral WAT and BAT metabolism, increased thermogenic markers and improved glucose metabolism, which is in line with the previous findings that CSN resection improves dysmetabolism by positively impacting insulin signaling and glucose uptake in visceral adipose tissue [4]. This reinforces the view that there is a connection between BAT and CB chemoreceptors [12] and provides evidence of a critical link between the CB that can drive metabolic improvement and decrease in weight gain by restoring WAT sensitivity to catecholamines and increasing BAT metabolism. Given that CB inputs are known to be integrated in the PVN [16] and the CSN denervation increases TH activity in the PVN in dysmetabolism (Figure 7), we can postulate that the link between the CB and BAT and WAT metabolism is mediated by integration of CB inputs at the PVN of the hypothalamus. Importantly, TH‐positive labeling in CSN‐resected animals was mainly located in the lateral parvocellular region that is known to be involved in the autonomic control [32], thus supporting the CB‐PVN‐sympathetic efferents‐adipose tissue link described here.

### 4.1 Strengths and Limitations

While the use of multiple methodologies supported by molecular data provided valuable insights into the mechanistic link between the carotid body and adipose tissue metabolism—with consistent results in both rat and mouse models, strengthening the validity of our hypothesis and the conservation of the mechanism—certain limitations must be considered. Specifically, our study relies on an early‐stage diabesity model induced by hypercaloric diets. Although this approach better mimic the type 2 diabetes progression in humans, it does not capture the later stages of the disease, as the severe hyperglycemia or β‐cell failure, which could be modeled using db/db or ob/ob mice or Zucker diabetic fatty rats [22]. Another limitation of our study was the approach used to modulate CB/CSN activity that was performed herein aiming the proof of concept of the potential of CB/CSN modulation as a therapeutic intervention for obesity and associated dysmetabolic state and the mechanism behind it. It is known that the CSN can regenerate over time [61], making its sectioning unsuitable as a long‐term therapeutic strategy in humans. Moreover, CSN cut is associated with several adverse effects as the loss of the responses to hypoxia and fluctuations in blood pressure [62]. Therefore, in the future, to fully explore its clinical applicability, alternative strategies—such as pharmacological interventions or CSN electrical modulation—must be investigated to assess their full therapeutic potential for obesity treatment.

## 5 Conclusions

In conclusion, this work identifies a critical circuit involving the CB, the hypothalamus, and its sympathetic efferents to the adipose tissue in the control of glucose and energy homeostasis. We identify that CB inputs govern a unique mechanism by which the hypothalamus and the SNS provide control of WAT and BAT metabolism not only by restoring sympathetic drive to the adipose tissue but also adjusting catecholaminergic signaling, favoring thermogenesis and energy expenditure.

## Author Contributions

**Bernardete F. Melo:** investigation, data curation, formal analysis, visualization, writing – original draft, writing – review and editing. **Joana F. Sacramento:** investigation, data curation, formal analysis, visualization, writing – original draft, writing – review and editing. **Julien Lavergne:** investigation, formal analysis, data curation, visualization. **Fátima O. Martins:** investigation, data curation, formal analysis, visualization. **Daniela Rosendo‐Silva:** investigation, formal analysis, data curation, visualization. **Clara Panzolini:** investigation, formal analysis, visualization, data curation. **Cláudia S. Prego:** data curation, formal analysis, visualization, investigation. **Aidan Falvey:** investigation, formal analysis, data curation, visualization. **Elena Olea:** investigation, formal analysis, data curation, visualization. **Paulo Matafome:** writing – review and editing, formal analysis, supervision, data curation, visualization, investigation. **Asuncion Rocher:** funding acquisition, resources, supervision, validation, formal analysis, writing – review and editing. **Jesus Prieto‐Lloret:** investigation, formal analysis, data curation, visualization. **Miguel C. Correia:** investigation, formal analysis, data curation, visualization. **Phillipe Blancou:** conceptualization, investigation, funding acquisition, methodology, validation, visualization, writing – review and editing, formal analysis, project administration, data curation, supervision, resources. **Silvia V. Conde:** conceptualization, methodology, investigation, validation, formal analysis, data curation, supervision, resources, project administration, writing – review and editing, visualization, funding acquisition, writing – original draft.

## Conflicts of Interest

The authors declare no conflicts of interest.

## Supporting information


**Table S1.** Plasma catecholamines levels and sympathetic nervous system (SNS).
**Figure S1.** Carotid sinus nerve (CSN) resection/denervation decreases respiratory responses to hypoxia.

## Data Availability

The data that support the findings of this study are available from the corresponding author upon reasonable request.
