# Supplementary material for: Reversal of Diabesity Through Modulating Sympathetic Inputs to Adipose Tissue Following Carotid Body Resection
Source: Acta Physiol (Oxf). 2025 Jun 24;241(7):e70074. doi: 10.1111/apha.70074 (PMC12186215; doi:10.1111/apha.70074)
Supplement: Supplementary file 1 — Table S1. Plasma catecholamines levels and sympathetic nervous system (SNS). Figure S1. Carotid sinus nerve (CSN) resection/denervation decreases respiratory responses to hypoxia. [file APHA-241-e70074-s001.pdf]

Supplemental data

Table S1 - Plasma catecholamines levels and sympathetic nervous system (SNS) index in obese dysmetabolic rats.

**Plasma Catecholamines (pmol/ml)**	**Without CSN resection**	**With CSN resection**
**Norepinephrine**		
NC	30.04±5.98	25.28±7.92
HF	65.52±15.83*	50.16±10.23
**Epinephrine**		
NC	41.17±5.97	46.23±11.94
HF	107.70±27.31*	38.22±5.77
**Dopamine + DOPAC**		
NC	1.93±0.18	2.30±0.67
HF	1.51±0.20	2.08±0.42
**SNS Index**	**Without CSN resection**	**With CSN resection**
NC	39.8±5.37	46.8±6.39
HF	58.0±5.22*	38.2±5.57$^{\#}$
**PNS Index**	**Without CSN resection**	**With CSN resection**
NC	-4.92±0.35	-5.6±0.13
HF	-4.60±0.26	-4.38±0.40

Data are mean values ± SEM of 6 - 11 animals. Two-Way ANOVA with Bonferroni multicomparison test. *p<0.05 comparing CTL - normal chow - vs HF groups; #p<0.05 comparing values with and without CSN resection.

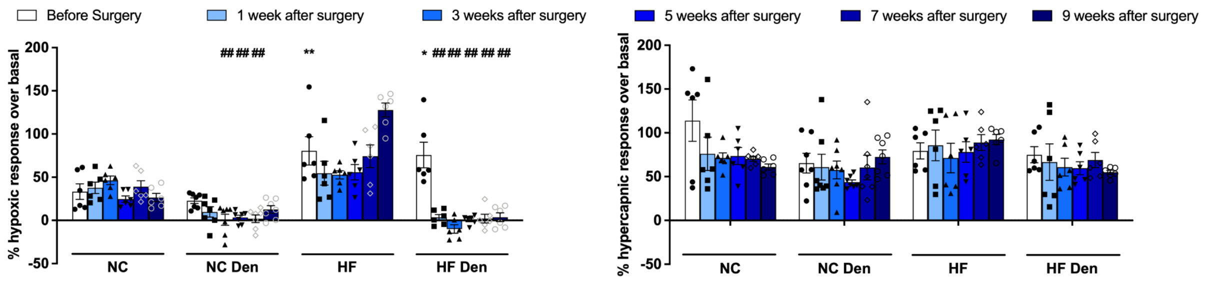

Figure S1 – Carotid Sinus Nerve (CSN) resection/denervation decreases respiratory responses to hypoxia in rats. A) respiratory responses to hypoxia; B) respiratory responses to hypercapnia; Protocol used consisted in animal acclimatization during 30 min followed by 10 min of normoxia (20% O_2 balanced N_2), 10 min of hypoxia (10% O_2 balanced N_2), 10 min of normoxia, 10 min of hypercapnia (20% O_2 + 5% CO_2 balanced N_2), and finally to 10 min of normoxia. Bars represent means ± SEM (n=9-15); Two-Way ANOVA with Bonferroni multicomparison test. *$p<0.05$, comparing NC and HF animals without CSN resection; $^{\#}p<0.05$ comparing groups before and after CSN resection.
